# P-1050. A Decade of *Pneumocystis jirovecii* Pneumonia: a Single-Center Retrospective Analysis in Both HIV and Non-HIV Populations

**DOI:** 10.1093/ofid/ofae631.1240

**Published:** 2025-01-29

**Authors:** Susana Ramos Oliveira, Clara Bacelar, Constança Azeredo, Maria João Gonçalves, Frederico Duarte, Eduarda Pena, Ricardo Correia de Abreu, Sofia Jordão

**Affiliations:** Unidade Local de Saúde de Matosinhos - Hospital Pedro Hispano, Porto, Porto, Portugal; Hospital Pedro Hispano, Porto, Porto, Portugal; Unidade Local de Saúde de Matosinhos - Hospital Pedro Hispano, Porto, Porto, Portugal; Hospital Pedro Hispano, Porto, Porto, Portugal; Hospital Pedro Hispano - Unidade Local de Saúde de Matosinhos, Matosinhos, Porto, Portugal; Hospital Pedro Hispano, Porto, Porto, Portugal; Hospital Pedro Hispano - Unidade Local de Saúde de Matosinhos, Matosinhos, Porto, Portugal; Hospital Pedro Hispano - Unidade Local Saúde Matosinhos, Matosinhos, Porto, Portugal

## Abstract

**Background:**

*Pneumocystis jirovecii* pneumonia (PJP) is a life-threatening opportunistic disease, commonly occurring in individuals with human immunodeficiency virus (HIV) or immunodepression. This study analyzes PJP cases in our local setting, detailing the epidemiological and clinical characteristics, as well as prognosis, and underlying diseases.

**Methods:**

Retrospective analysis of adult inpatient PJP cases (2014-2023). Diagnosis was presumptive (epidemiological, clinical, and imaging findings), or confirmed microbiologically. Statistical analysis utilized the chi-square test.

**Results:**

Forty-two patients were identified: 66% male, median age of 47.5 years [25-83]. Seventy-four percent were HIV positive, with a median CD4+ count of 35 [1-347]. All had detectable HIV viral load, except one (suppressed for years, but with a new diagnosis of neoplasm). Non-HIV patients (26%) underlying diseases included hematological (n=6) or solid-organ malignancies (n=1), autoimmune diseases under corticosteroid therapy (n=2), pulmonary interstitial disease (n=1), and granulomatous systemic disease under investigation (n=1). Eighty-six percent had peripheral lymphopenia at diagnosis, and only one patient was receiving regular prophylaxis. The incidence of non-HIV patients significantly increased in the latter 5 years (5.5% vs 42%, p=0.008). Diagnosis was confirmed by positive direct antibody fluorescence staining in half of the patients. No molecular biology techniques or B-D-glucan assays were available. Seventeen (40%) patients required admission to level II or III units, of which 23% required invasive mechanical ventilation and 35% high-flow oxygen. Treatment included cotrimoxazole (n=40) and atovaquone (n=2). One-third of patients had cytomegalovirus reactivation, requiring antiviral therapy. Non-HIV patient mortality was significantly higher than HIV patients (45% vs 13%, p=0.023). Fifteen percent of survivors were readmitted within 30 days.

**Conclusion:**

The epidemiology of PJP is evolving, with a noticeable increase in non-HIV cases, which are associated with significantly higher mortality despite appropriate treatment. Authors emphasize the importance of identifying new risk groups for chemoprophylaxis to improve prognosis.

**Disclosures:**

**All Authors**: No reported disclosures

